# Dataset for phenotypic classification of genetic modifiers of smoothened and Hedgehog

**DOI:** 10.1016/j.dib.2016.02.076

**Published:** 2016-03-04

**Authors:** Suresh Marada, Ashley Truong, Stacey K. Ogden

**Affiliations:** a Department of Cell and Molecular Biology, St. Jude Children׳s Research Hospital, Memphis, TN 38105, USA; b Rhodes College Summer Plus Program, Rhodes College, Memphis, TN 38112, USA

## Abstract

This data article includes supporting information for the research article entitled “The Small GTPase Rap1 is a Modulator of Hedgehog Signaling” [1]. Drosophila wing phenotypes induced by expression of a dominant negative Smoothened (Smo) mutant were cataloged into five distinct classes. Class distributions observed following expression of dominant negative Smo in control and sensitized backgrounds were quantified to serve as references for strength of phenotypic modification. Shifts in class distribution of Hedgehog (Hh) wing phenotypes resulting from introduction of loss-of-function alleles of select Ras family G protein genes and the Hh pathway regulators Fused and Suppressor of Fused are shown.

**Specifications Table**

**Table:** 

Subject area	Biology
More specific subject area	Signal transduction
Type of data	Images and graphs
How data was acquired	Zeiss Stemi 2000 with ICc 3 camera
Data format	Raw and analyzed
Experimental factors	Drosophila crosses
Experimental features	Light microscopy, phenotype classification and quantification
Data source location	St. Jude Children׳s Research Hospital, Memphis, TN
Data accessibility	Data are supplied with this article

## Value of the data

● Establishes classes for Hh loss-of-function wing phenotypes that can be used to quantify results from genetic modifier screens.
● Quantifies the effect of a mutant allele of a negative regulator of Hh signaling on a loss-of-function phenotype to serve as a reference for scoring negative pathway regulators identified through modifier screens.
● Quantifies effect of a mutant allele of a positive regulator of Hh signaling on a gain-of-function background to serve as a reference for scoring positive pathway regulators in modifier screens.
● Quantifies the effect of loss-of-function alleles of select Ras family monomeric G proteins on Hh wing phenotypes.

## 1 Data

**Fig. 1:**
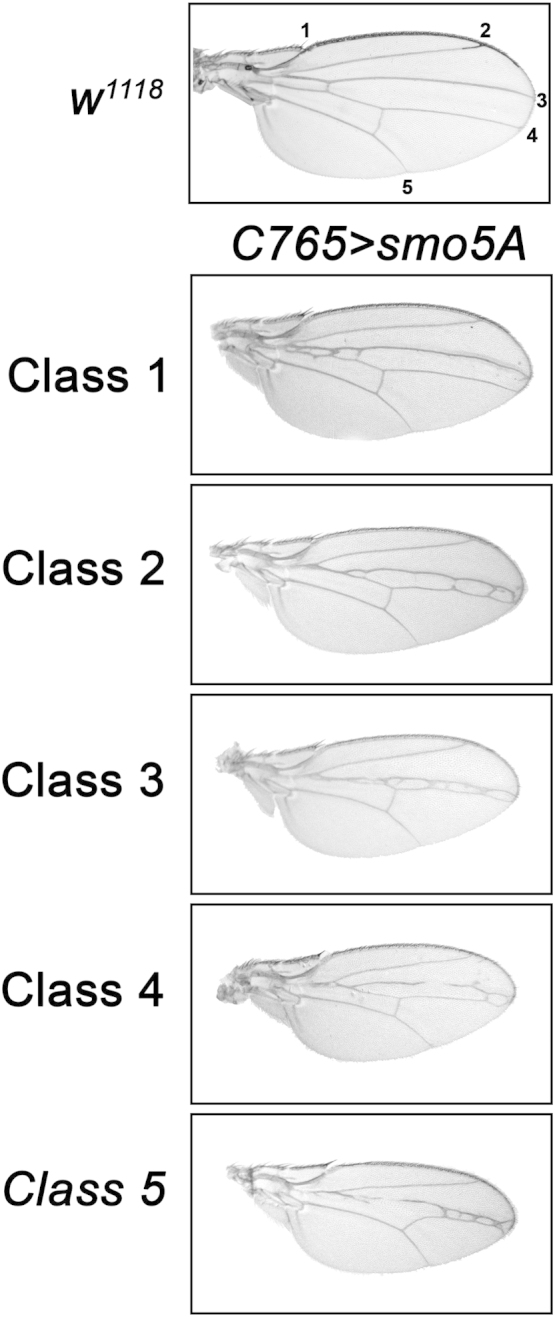
*smo5A* classes**.** Wing phenotypes induced by *C765>smo5A* were classified based upon phenotypic severity. Representative wings for each class are shown.

**Fig. 2:**
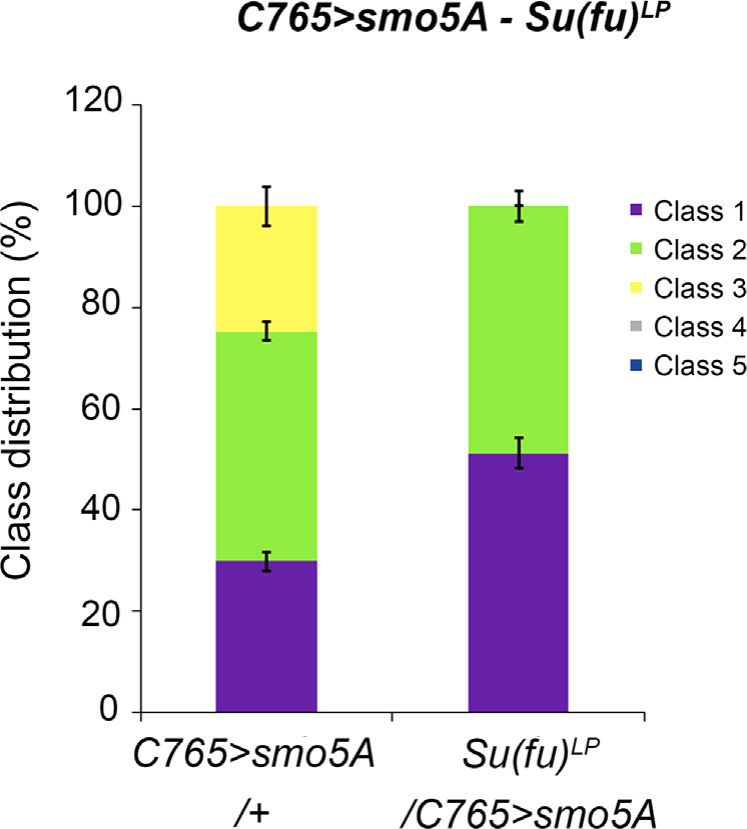
*smo5A* class distribution is shifted by *Su*(*fu*)^*LP*^ mutation. Percent distribution of Smo5A classes observed in ~150 male progeny across three independent crosses is shown. Error bars indicate standard error of the mean (s.e.m.).

We present results from Drosophila genetic modifier screens that can be used to score genetic interactions that impact Hh signal transduction as in [1]. Wing phenotypes induced by dominant negative Smo5A protein [2] were cataloged into classes based upon severity of fusions between longitudinal veins 3 and 4 (LV3/LV4, Fig. 1). Classes were used to quantify phenotypic modification resulting from introduction of a loss-of-function allele of the negative pathway regulator Suppressor of Fused (*Su*(*fu*)^*LP*^, Fig. 2).

**Fig. 3:**
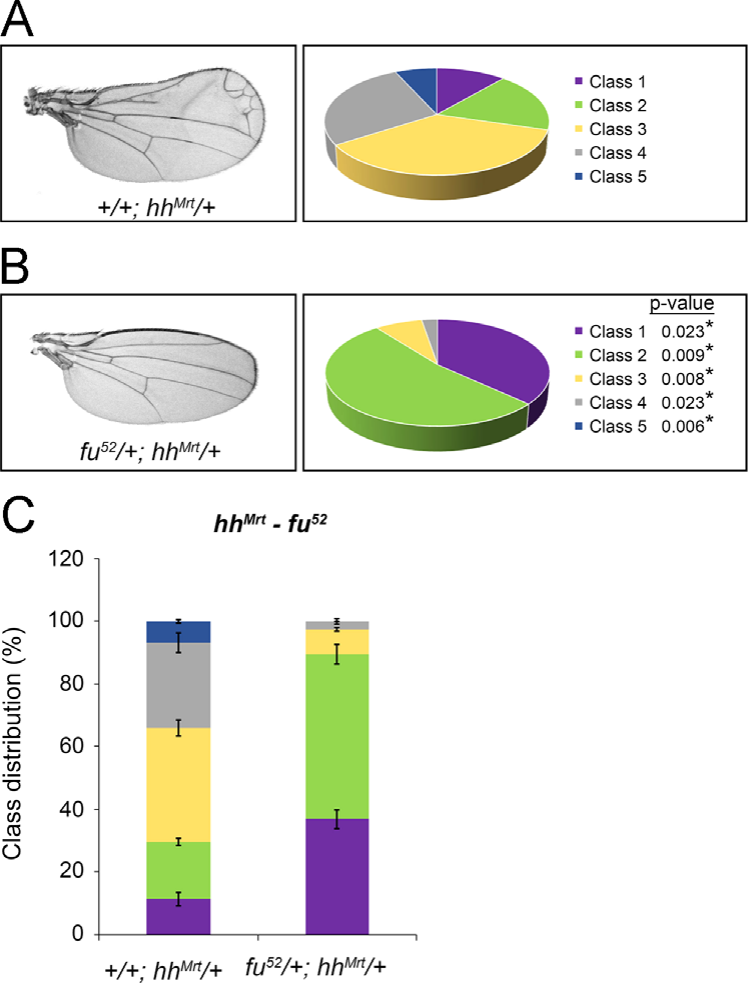
*hh*^*Mrt*^ class distribution is shifted by *fu* mutation. *fu*^*52*^ was introduced into the *hh*^*Mrt*^ background. Percent class distribution of *hh*^*Mrt*^ classes observed in ~150 male progeny across three independent crosses is shown by pie chart and bar graph. Significance of percent shift in each class is indicated. Error bars indicate s.e.m.

**Fig. 4:**
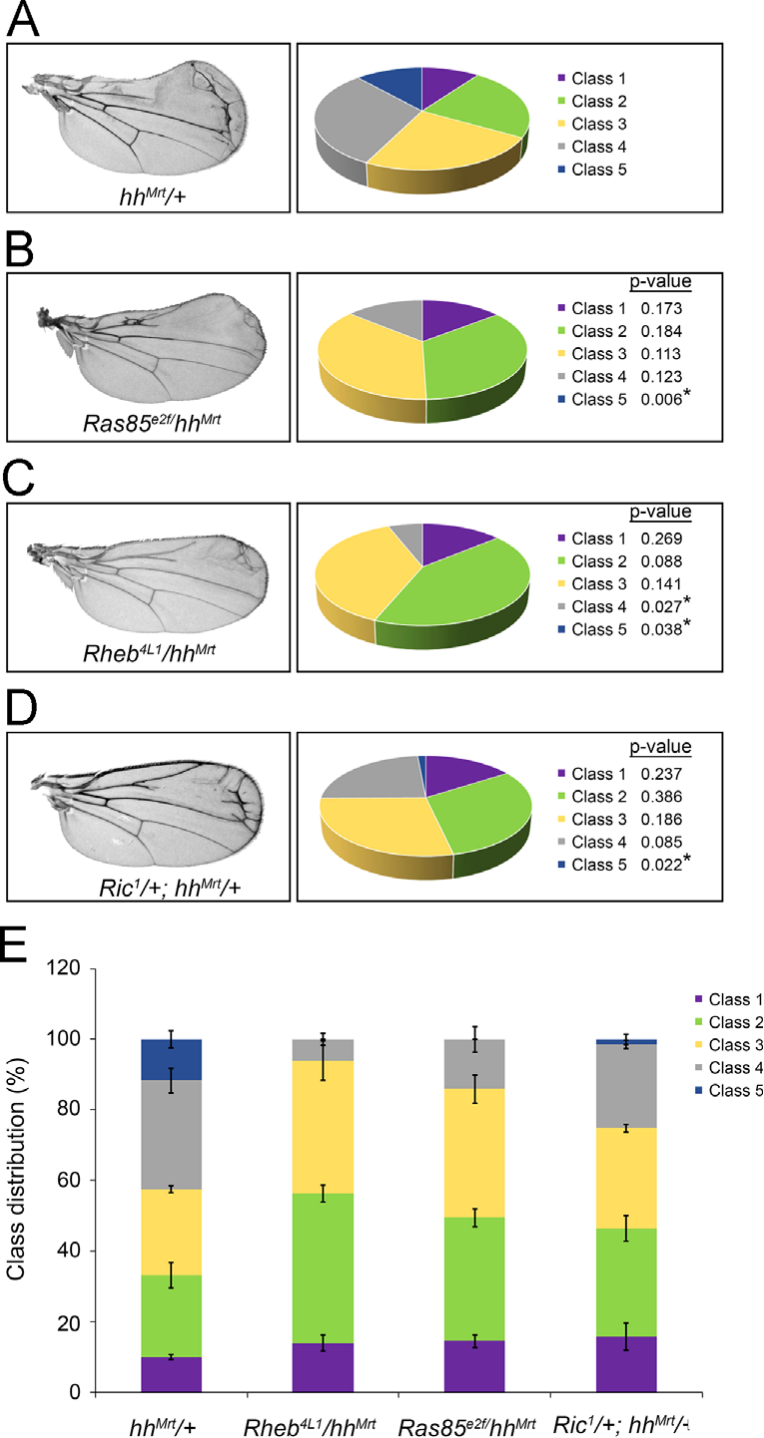
Ras subfamily GTPase effects on *hh*^*Mrt*^. The indicated Ras family mutant alleles were introduced into the *hh*^*Mrt*^ background. The percent of the experimental population falling into each phenotypic class is indicated by pie charts (A–D) and plotted with error bars (E). Class distribution from ~75 male progeny over 3 independent crosses is shown. Wings representative of the most predominant class are shown for each. Significance of percent shift in each class is indicated. Error bars indicate s.e.m.

Loss-of-function alleles of the known positive pathway effector Fused (*fu*^*52*^) or select Ras family small G proteins were introduced into the *hh*^*Mrt*^ gain-of-function background [3], [4] and modification of phenotypic class distribution was quantified (Figs. 3 and 4).

## 2 Experimental design, materials and methods

A transgene encoding the Smo5A mutant with S/T to A changes of five essential PKA phosphorylation sites (Smo5A) was expressed in the developing wing imaginal disc using the *C765-GAL4* driver [2]. Mutant alleles of the indicated genes were crossed into *C765>smo5A* or *hh*^*Mrt*^ backgrounds using standard techniques [1]. Wings from ~75–100 male progeny were analyzed from each class to quantify phenotypic modification.
